# Supplementary material for: WordCommentsAnalyzer: A windows software tool for qualitative research
Source: F1000Res. 2018 Sep 4;7:536. Originally published 2018 May 3. [Version 2] doi: 10.12688/f1000research.14819.2 (PMC6137411; doi:10.12688/f1000research.14819.2)
Supplement: Supplementary file 3 [file f1000research-7-17614-s0002.tgz › b2a79496-4ada-47c4-9682-a99ce867836d.zip!/Eight Iranian Health Journals/Journal of Health System Research _40.docx]

© Journal of Health System Research . This work is licensed under CC BY-NC 4.0 (https://creativecommons.org/licenses/by-nc/4.0/).

Volume & Issue: دوره 7، شماره 5؛1390

Title: بررسي مسؤول مداخلات از طریق لوح فشرده چند رسانه‌ای بر روی شیوه زندگی جوانان در طی سال‌های 86-1382 (پروژه جوانان طرح ملی قلب سالم اصفهان)

http://hsr.mui.ac.ir/index.php/jhsr/article/view/311

مقدمه: از آن جايي كه عادات زندگي از دوران نوجواني شكل مي‌گيرند، نوجوانان هدف اصلي برنامه‌هاي آموزشي مي‌باشند. از سوی دیگر استفاده گسترده جوانان و نوجوانان از رسانه‌هاي جمعي دليل توجه بيشتر به نقش این وسایط در جامعه به خصوص در حيطه موضوعات مرتبط با سلامت و شیوه زندگي در سال‌هاي اخير مي‌باشد. به همین دلیل، این مطالعه به بررسی تأثیر مداخلات برنامه قلب سالم اصفهان، از طریق لوح فشرده چند رسانه‌ای در جوانان 25-19 سال مراجعه کننده به استان اصفهان در مقایسه با شهرستان اراک می‌پردازد. مواد و روش‌ها: مطالعه حاضر از نوع مداخله‌ای بوده، که جمعیت مورد مطالعه جوانان 25- 9 سال شهر اصفهان و نجف‌آباد شرکت کننده در کلاس‌های مختلف اصفهان و شعبات آن بودند. معیارهای ورود به مطالعه سن 25-19 سال و امکان استفاده از لوح‌های فشرده بود که افراد شرکت‌کننده در کلاس‌های آموزشی مختلف در صورت تمایل و امضای موافقت‌نامه در این مطالعه وارد شده، پرسش‌نامه استاندارد شده و استفاده شده قبلی توسط برنامه طرح ملی قلب سالم اصفهان برای افراد شرکت‌کننده قبل و بعد از مطالعه تکمیل شد. آموزش‌های CD بر اساس آموزش اجتناب از مصرف غذاهای فست‌‌فودی و دخانیات و استرس و زندگی ناسالم بود. افراد شرکت‌کننده به دو گروه شاهد (شهرستان اراک) و مورد مداخله (شهرستان اصفهان و نجف‌آباد) تقسیم شدند. نتایج: میانگین مصرف نوشابه‌های گاز‌دار و غذاهای آماده در جمعیت مورد مداخله در سال‌های مداخله کاهش بیشتری نسبت به جمعیت رفرانس داشته است. درصد افراد با استرس بالا در جمعیت مورد مداخله نیز کاهش بیشتری نسبت به جمعیت رفرانس نشان داد. همچنین در جمعیت رفرانس درصد کل افراد سیگاری افزایش داشت، ولی در جمعیت مورد مداخله کاهش یافته بود. همچنین فعالیت فیزیکی در جمعیت مورد مداخله افزایش اندکی داشته است؛ در حالی که میزان فعالیت فیزیکی در جمعیت رفرانس کمتر شده بود. بحث و نتیجه‌گیری: نتایج مثبت حاصل از مداخلات برنامه قلب سالم اصفهان، از طریق لوح فشرده چند رسانه‌ای در جوانان 25-19 سال مراجعه کننده به هلال‌ احمر استان اصفهان، نشان می‌دهد که به کارگیری رسانه‌های جمعی مثل لوح فشرده جهت اصلاح شیوه زندگی برای پیش‌گیری از بیماری‌های غیر واگیر می‌تواند مؤثر باشد.

Title: کارایی نانو زئولیت اصلاح شده با سورفکتانت کاتیونی در حذف پیش‌سازهای محصولات ثانوی گندزدایی از آب آشامیدنی

http://hsr.mui.ac.ir/index.php/jhsr/article/view/312

مقدمه: مواد آلی طبیعی نظیر اسید هیومیک عامل اصلی تولید محصولات ثانویه گندزدایی می‌باشد. هدف این مطالعه تعیین کارایی نانو زئولیت اصلاح شده با سورفکتانت در حذف اسید هیومیک از آب بود. روش‌ها: اصلاح نانو زئولیت کلینوپتیلولایت توسط سورفکتانت کاتیونی هگزا دسیل تری متیل آمونیوم برماید با دوزهای 5/0، 1، 3، 5، 7 و g/l 10 انجام گرفت. در ابتدا و انتهای فرایند جذب، غلظت اسید هیومیک به همراه کدورت و pH مورد آزمایش قرار گرفت. یافته‌ها: آزمون آماری ANOVA یک‌ طرفه، بین دوز سورفکتانت کاتیونی و غلظت اسید هیومیک حذف شده، ارتباط معنی‌داری نشان داد (001/0 P <)؛ به طوری که با افزایش دوز سورفکتانت کاتیونی، نسبت‌های کدورت اولیه به کدورت نهایی و 0-254UV (میزان جذب نور در طول موج 254UV در نمونه اولیه) به 254UV (نهایی) دارای تغییراتی بود. در دوز 7 و 10 گرم در لیتر سورفکتانت کاتیونی، بیشترین راندمان حذف اسید هیومیک به ترتیب 96 و 86 درصد با کمترین تغییرات در مقدار pH و کدورت در درجه حرارت اتاق (C° 2 ± 20) به دست آمد. نتیجه‌گیری: نتایج این مطالعه نشان داد که اصلاح نانو زئولیت برای حذف پیش‌سازهای محصولات ثانوی گندزدایی ضروری می‌باشد؛ چرا که این ترکیبات به علت دارا بودن بار سطحی منفی، توانایی جذب آنیون‌ها را ندارند. همچنین مصرف سورفکتانت کاتیونی با دوز g/l 7 و راندمان حذف 96 درصد با داشتن pH منطبق بر نقطه ایزوالکتریک می‌تواند به عنوان غلظت بهینه مورد استفاده قرار گیرد.

Title: بررسي ميزان آگاهي، نگرش و عملكرد مردم منطقه جرقویه- رامشه در زمينه مديريت مواد زايد جامد

http://hsr.mui.ac.ir/index.php/jhsr/article/view/313

مقدمه: مدیریت مواد زاید جامد از هشت عنصر کاهش در مبدا، تولید، ذخیره در محل، جمع‌آوری، حمل و نقل، پردازش و بازیافت، دفع و مراقبت‌های پس از دفع تشکیل شده است. مرحله تولید در فرایند مدیریت مواد زاید نقش اساسی دارد و مردم به عنوان تشکیل‌دهندگان اولین حلقه زنجیره با آگاهی و نگرش صحیح نسبت به این موضوع می‌توانند موجب عملکرد صحیح‌تر و مدیریت کاراتر سیستم گردند. اين پژوهش با هدف بررسي آگاهي، نگرش و عملكرد مردم منطقه رامشه در زمينه مديريت مواد زايد جامد تدوين و اجرا گرديد. روش‌ها: این پژوهش از نوع توصیفی– تحلیلی است که به روش مقطعی انجام شد. جامعه مورد بررسی 90 نفر از منطقه رامشه شهرستان اصفهان بود که توسط پرسش‌نامه خود ایفا مورد پرسش قرار گرفتند. به منظور تحلیل داده‌ها از نرم‌افراز 5/11SPSS استفاده گردید. یافته‌ها: میانگین نمره آگاهی در این بررسی 2/1 ± 05/8 (از 14 امتیاز) بود که متوسط به بالا ارزیابی گردید. میانگین نمره نگرش 3/2 ± 52/2 به دست آمد (صفر به منزله بی‌تفاوت بودن است) که نشان داد که جامعه مورد مطالعه از نگرش مثبتی برخوردار است. 5/38 درصد از مردم دفن بهداشتی و 7/33 درصد بازیافت را بهترین روش دفع زباله معرفی کردند. 64 درصد افراد، جمع‌آوری زباله را دو بار در هفته لازم دانستند. همچنین 3/38 درصد افراد با بیشترین فراوانی معتقد بودند که زباله‌ها بهتر است بین ساعت 9-7 صبح جمع‌آوری شود. 3/32 درصد مردم با فروش زباله به افراد دوره گرد به طور نسبی مخالف و 3/29 درصد کاملاً مخالف بودند. نتیجه‌گیری: با توجه به یافته‌های این تحقیق، بين سطح آگاهي با شغل و همچنين سطح تحصيلات رابطه معنی‌دار مشاهده گرديد. با توجه به اين كه آگاهي افراد در رابطه با موضوع مورد بررسي به نسبت مناسب بود، بايستي اين موضوع كه چه عواملي باعث شده است كه افراد از عملكرد پاييني برخوردار باشند مورد توجه قرار داد. همچنین برنامه‌ريزي مناسب براي بهبود و ارتقای اين امر صورت داد. به نظر می‌رسد که وجود یک برنامه بازیافت قوی و قابل اجرا به نحوی که حداکثر مشارکت مردمی را به دنبال داشته باشد، برای بهبود وضعیت مدیریت مواد زاید جامد مؤثر باشد.

Title: بررسی آگاهی، نگرش و عملکرد دانشجویان دانشگاه‌های شهر اصفهان نسبت به خوددرمانی با دارو

http://hsr.mui.ac.ir/index.php/jhsr/article/view/314

مقدمه: خوددرمانی یکی از شایع‌ترین اعمال در سراسر جهان است. آمار و اطلاعات گویای افزایش شیوع مصرف خودسرانه داروها در کشور ایران است. از این رو این مطالعه با هدف بررسی آگاهی، نگرش و عملکرد دانشجویان دانشگاه‌های شهر اصفهان در خصوص خوددرمانی انجام گرفت. روش‌ها: پژوهش حاضر یک مطالعه توصیفی، تحلیلی و مقطعی است که 600 نفر از دانشجویان دانشگاه‌های اصفهان در آن شرکت نمودند. با توجه به وجود 3 دانشگاه دولتی در سطح شهر اصفهان، نمونه‌گیری در مطالعه حاضر به صورت چند مرحله‌ایی و بر اساس تعداد کل دانشجویان هر دانشگاه صورت گرفت. در پژوهش، اطلاعات به وسیله پرسش‌نامه محقق ساخته جمع‌آوری گردید. اعتبار پرسش‌نامه با استفاده از روش اعتبار محتوا و پایایی ابزار نیز از طریق همسانی درونی (7/0) تأیید شد. پس از گردآوری اطلاعات، داده‌ها با استفاده از نرم‌افزار 15SPSS و آزمون‌های آماری همبستگی پیرسون، t-test مستقل و آنالیز واریانس، مورد تجزیه و تحلیل قرار گرفت. یافته‌ها: میانگین نمره آگاهی (9/17 ± 93/83) و عملکرد صحیح (49/6 ± 58/97) دانشجویان در سطح بالایی قرار داشت اما نگرش دانشجویان در زمینه خوددرمانی در سطح متوسطی قرار داشت. در این مطالعه 2/14 درصد از دانشجویان در طی 2 ماه گذشته حداقل در مورد یک بیماری اقدام به مصرف خودسرانه دارو نمودند. بیشترین فراوانی خوددرمانی به تفکیک نوع بیماری شامل سرماخوردگی و به تفکیک نوع دارو، شامل داروهای مسکن بود. عمده‌ترین علل خوددرمانی نیز تجربه قبلی از بیماری و در دسترس بودن داروها و مهم‌ترین عامل پیشگیری‌کننده در زمینه خوددرمانی، عدم اعتقاد به خوددرمانی بود. نتیجه‌گیری: با توجه به یافته‌های پژوهش، ارایه آموزش‌هایی به دانشجویان در جهت اصلاح نگرش آنان ضروری به نظر می‌رسد تا علاوه به بهبود عملکرد آنان از تأثیر این نگرش به عنوان افراد تحصیل کرده در جامعه جلوگیری شود.

Title: بررسی اثر مصرف شیر سویا روی فاکتورهای التهابی و قلبی- عروقی زنان بزرگسال دارای اضافه وزن و چاقی

http://hsr.mui.ac.ir/index.php/jhsr/article/view/315

مقدمه:جایگزینی شیر سویا در رژیم غذایی می‌تواند اثرات مهمی روی فاکتورهای التهابی و قلبی- عروقی زنان غیر یائسه دارای اضافه وزن و چاق داشته باشد. در این مطالعه اثرات شیر سویا در مقایسه با شیر گاو روی فاکتورهای التهابی و قلبی- عروقی زنان بزرگسال دارای اضافه وزن و چاقی ارزیابی شد. روش‌ها این مطالعه، مطالعه کارآزمایی بالینی متقاطع بر روی 24 بیمار بود. تمامی بیماران از رژیم کاهش وزن استفاده کردند. در این بررسی 2 دوره مداخله 4 هفته‌ای (یک دوره رژیم کاهش وزن همراه با شیر سویا و دوره دیگر رژیم کاهش وزن همراه با شیر گاو) و یک دوره washout 2 هفته‌ای وجود داشت. در دوره شیر سویا تنها یک لیوان (240 سی‌سی) شیر سویا جایگزین یک لیوان (240 سی‌سی) شیر گاو شد. یافته‌ها در طی دوره شیر سویا LDL و دور کمر به طور چشمگیری کاهش یافت. میانگین درصد تغییرات برایLDL  در دوره شیر سویا 85/3±  22/11- و در دوره شیر گاو 82/2 ± 18/1- (01/0 =P ) و برای دور کمر در دوره شیر سویا 51/0  ±79/3- و در دوره شیر گاو 55/0 ± 78/1- (02/0 =P ) بود. اما فاکتورهای التهابی، آنزیم‌های کبدی، فشار خون، بقیه فاکتورهای لیپیدی وشاخص‌های گلیسمیک بعد از مصرف دوره شیر سویا در مقایسه با دوره مصرف شیر گاو تغییر چشمگیری پیدا نکرد. نتیجه‌گیری رژیم غذایی همراه با شیر سویا می‌تواند اثرات مهمی در کاهش LDL و محیط دور کمر در زنان غیر یائسه دارای اضافه وزن و چاقی حتی در یک مداخله کوتاه مدت داشته باشد. به هر حال رژیم همراه با شیر سویا تأثیر چشمگیری روی فاکتورهای التهابی، آنزیم‌های کبدی، فشار خون، سطح فیبرینوژن خون و شاخص‌های گلیسمیک نداشت.

Title: بررسی اثرات فضاهای شهری بر ناهنجاری‌های محیطی و رفتاری شهروندان منطقه 12 تهران

http://hsr.mui.ac.ir/index.php/jhsr/article/view/316

مقدمه: گذار جوامع سنتي به مدرن، همواره يك پيامد مهم در بر دارد و آن گسترش روزافزون پديده شهرنشيني است. زندگي مدرن و شهرنشيني به همراه خود شرايط و پديده‌هايي را به همراه مي‌آورد كه مي‌تواند بر روي مفهوم «سلامت اجتماعي و فردی افراد» مؤثر واقع شود. این پدیده باعث شده است که بسیاری از نظریه‌پردازان شهری به خصوص مکتب شیگاکو به بررسی پدیده ناهنجاری محیطی بر سلامت شهروندان توجه خاصی مبذول نمایند. پژوهش حاضر به بررسی اثرات فضاهای شهری بر پدیده ناهنجاری‌های محیطی و رفتاری جوانان منطقه 12 تهران پرداخته است. روش: روش تحقیق، پیمایشی بوده، برای سنجش متغیرهای مستقل و وابسته، تعداد 200 نفر از جوانان 30-15 ساله منطقه 12 تهران به شیوه نمونه‌گیری تصادفی ساده انتخاب شدند و ابزار گرد‌آوری اطلاعات پرسش‌نامه محقق ساخته بود. یافت‌ها:‌ آزمون 2χ نشان داد که بین فضای شهری، آسایش محیطی و آلودگی‌های صوتی و تصویری (شامل: سیمای شهر، آشفتگی منظر، وجود صنایع آلوده کننده) نابهنجار، ‌و میزان رفتارهای شهروندان (ارتکاب جرم و ابتلا به رفتارهای پرخطر) رابطه معنی‌داری وجود دارد.‌ بحث و نتیجه‌گیری: هر قدر پدیده فضاهای شهری چه در بعد و محتوا دارای کارکرد منفی‌تری باشند، به همان اندازه سلامت روانی و اجتماعی شهروندان به خصوص جوانان را تحت تأثیر قرار می‌دهند.

Title: بررسی کیفیت زندگی در بیماران مبتلا به مولتیپل اسکلروزیس شهر اصفهان

http://hsr.mui.ac.ir/index.php/jhsr/article/view/317

مقدمه: مولتیپل اسکلروزیس (Multiple sclerosis، MS) یک بیماری مزمن و پیشرونده دستگاه عصبی مرکزی است که به میلین اعصاب مغز و نخاع آسیب می‌رساند و باعث بروز عوارض متعددی در بیماران مبتلا می‌گردد. ناتوانی‌های فیزیکی و تغییرات روانی می‌تواند بر روی عزت نفس و کیفیت زندگی بیماران مبتلا به MS تأثیر گذارد. این مطالعه با هدف ارزیابی کیفیت زندگی بیماران مبتلا به MS شهر اصفهان طراحی و اجرا گردید. روش‌کار: این پژوهش به صورت مقطعی انجام شد. جامعه پژوهش، بیماران عضو انجمن MS اصفهان تشکیل دادند. نحوه نمونه‌گیری به صورت طبقه‌ای 2 مرحله‌ای، خوشه‌ای و متناسب با حجم نمونه انجام گرفت که از بین 4250 نفر بیمار مبتلا در اصفهان، 251 نفر مراجعه‌کننده به انجمن MS و بیمارستان الزهرا (س) و بر اساس نوع بیماری وارد مطالعه شدند. اطلاعات دموگرافیک آن‌ها نیز ثبت شد. روش گردآوری اطلاعات به صورت خودگزارشی و با استفاده از پرسش‌نامه اختصاصی کیفیت زندگی بیماران مولتیپل اسکلروزیس (Multiple sclerosis quality of life، 54MSQOL-) بود که در مجموع حاوی 54 سؤال می‌باشد. یافته‌ها: تقریباً 50 درصد بیماران زن و 50 درصد مرد و دامنه سنی بیماران 61-17 سال (10/10 ± 34/36) بود. میانگین امتیازات کیفیت زندگی در دو بعد کلی سلامت جسمانی 69/15 ± 18/47 و سلامت روانی 46/15 ± 95/44 به دست آمد. کمترین نمره در بین دوازده خرده مقیاس مربوط به محدودیت نقش مرتبط با مشکلات جسمی (4/23 ± 5/43) و بیشترین نمره مربوط به عملکرد اجتماعی (7/14 ± 4/54) بود. بین سن، مدت زمان ابتلا و نوع بیماری با دو بعد کیفیت زندگی رابطه معکوس وجود داشت. بین سطح تحصیلات با دو بعد کیفیت زندگی رابطه مستقیم مشاهده شد (001/0 P <). آزمون همبستگی پیرسون ارتباط مستقیم بین ابعاد کیفیت زندگی با یکدیگر نشان داد (001/0 P <). نتیجه‌گیری: به طور کلی میانگین نمره کیفیت زندگی بیماران کمتر از حد متوسط بود. با توجه به ارتباط عوامل شخصی با كيفيت زندگي بیماران مبتلا به MS، آنچه جهت ارتقای كيفيت زندگي آنان مهم به نظر مي‌رسد، رویکرد بین بخشی به مسایل و مشكلات سلامتي و انجام مداخلات لازم در عوامل تأثیرگذار بر کیفیت زندگی این بیماران می‌باشد.

Title: بررسی میزان اعتیاد به اینترنت در دانشجویان دانشگاه پیام‌نور اصفهان واحد کهندژ

http://hsr.mui.ac.ir/index.php/jhsr/article/view/318

مقدمه: کاربردهای فراوان اینترنت و جذابیت‌های آن سبب شده در سال‌های اخیر پدیده‌ای با عنوان "اعتیاد به اینترنت" ظهور کند. اینترنت به خودی خود ابزار بی‌ضرری است اما استفاده بیش از حد و نادرست از آن، خطر اعتیاد به اینترنت را دنبال دارد. مطالعات انجام شده بر روی دانش‌آموزان و دانشجویان نشان‌دهنده این امر است که میزان استفاده از اینترنت، همچنین میزان بروز و شیوع اعتیاد به اینترنت در حال افزایش است. بنابراین این پژوهش با هدف تعیین میزان اعتیاد به اینترنت در دانشجویان دانشگاه پیام‌نور اصفهان واحد کهندژ صورت گرفت. روش‌کار: این پژوهش از نوع توصیفی- مقطعی است که در 96 نفر از دانشجویان دانشگاه پیام‌نور صورت گرفت. ابزار گردآوری داده‌ها پرسش‌نامه یانگ بود که در 3 بخش تنظیم گردید. بخش اول اطلاعات دموگرافیک، بخش دوم دلایل استفاده از اینترنت و بخش سوم شامل آزمون اعتیاد به اینترنت بود. پس از کسب رضایت آگاهانه از دانشجویان، پرسش‌نامه توسط آنان تکمیل گردید. اطلاعات وارد نرم‌افزار آماری 5/11SPSS شد. برای تجزیه و تحلیل داده‌ها از آزمون‌های همبستگی پیرسون، t-test، همبستگی اسپیرمن و من ویتنی داده‌ها جهت استفاده گردید. در نهایت وضعیت کاربران مورد مطالعه به صورت کاربر طبیعی، کاربر با اعتیاد خفیف و اعتیاد شدید مشخص گردید. یافته‌ها: در این مطالعه 7/91 درصد دانشجویان کاربر طبیعی بودند و 3/8 درصد آنان اعتیاد خفیف به اینترنت داشتند. بیشترین مورد استفاده از اینترنت در دانشجویان، استفاده از موتورهای جستجوگر و دانلود فایل بود. بین اطلاعات دموگرافیک با اعتیاد به اینترنت اختلاف معنی‌داری مشاهده نشد، اما بین مدت زمان استفاده و اعتیاد به اینترنت رابطه آماری معنی‌داری مشاهده گردید. همچنین 4/8 درصد دانشجویان عنوان کردند که به خاطر اینترنت دچار افت تحصیلی شدند.   نتیجه‌گیری: به نظر می‌رسد که اقداماتی از قبیل آموزش افراد در جهت استفاده صحیح از اینترنت و همچنین آموزش خانواده‌ها بتواند راه حل مناسبی جهت پیشگیری از اعتیاد به اینترنت در دانشجویان باشد.

Title: بررسی وضعیت آلودگی صوتی در شهر اصفهان

http://hsr.mui.ac.ir/index.php/jhsr/article/view/319

مقدمه: به منظور پیشگیری از اثرات آلودگی صوتی و دسترسی به استانداردهای توصیه شده، اندازه‌گیری آلودگی صوتی در شهرها امری ضروری است. از این رو این پژوهش با هدف بررسی میزان آلودگی صوتی در شهر اصفهان انجام گرفت. روش‌ها: در این مطالعه تعداد 19 ایستگاه سنجش میزان صوت که در نقاط کلیدی و پر رفت و آمد شهر اصفهان بود، بر اساس معیارهای موجود انتخاب شد. با استفاده از دستگاه صداسنج مدل 440CEL-، پارامترهای محیطی صدا شامل 10L، 50L، 90L، 95L، 99L، Leq،SEL ،SPL ،Lmin ، Lmax و  Lpeakاندازه‌گیری شد. برای محاسبه شاخص‌های ترافیکی آلودگی صوتی NPL و TNI از فرمول مربوطه استفاده گردید. اندازه‌گیری‌ها در دو نوبت صبح و بعد از ظهر با فواصل زمانی نیم ساعت انجام شد. یافته‌ها: حداکثر مقدار تراز معادل صدا (Leq) در نوبت صبح مربوط به چهارراه پل فردوسی به مقدار 3/78 دسی‌بل و در نوبت بعد از ظهر مربوط به میدان جمهوری به مقدار 6/79 دسی‌بل بود. میانگین و انحراف معیار مقادیر اندازه‌گیری شده Leq در نوبت صبح به ترتیب 5/74 و 78/2 و در نوبت بعد از ظهر 6/72 و 43/4 بود. مقایسه میانگین مقادیر Leq صبح و بعد از ظهر با عدد استاندارد اختلاف معنی‌داری (001/0 P <) را نشان داد. به عبارت دیگر مقدار Leq بالاتر از عدد استاندارد است. همچنین بین مقادیر محاسبه شده NPL و TNI نوبت صبح و نوبت بعد از ظهر رابطه مستقیمی وجود داشت (ضریب همبستگی پیرسون = 877/0 و 001/0 P <). نتیجه‌گیری: با توجه به نتایج به دست آمده مشخص شد که شهر اصفهان از آلودگی صوتی بالایی برخوردار است. در اکثر موارد نیز از استاندارد سازمان حفاظت محیط زیست ایران بالاتر است.

Title: اثربخشي مداخله آموزشي مبتني بر الگوي پرسيد بر سطح استرس (تنش) سالمندان مراجعه‌كننده به کانون‌های جهان دیدگان شهر تهران

http://hsr.mui.ac.ir/index.php/jhsr/article/view/320

مقدمه: با توجه به آسيب‌پذيري سالمندان در برابر اثرات منفي عوامل استرس‌زا و جهت كنترل اين عوامل، مطالعه حاضر با هدف تعيين تأثير مداخله آموزشي مبتني بر الگوي PRECEDE بر سطح استرس سالمندان صورت گرفت. روش‌ها: در اين مطالعه نيمه تجربي تعداد 94 سالمند مراجعه‌ کننده به کانون‌های جهان ‌دیدگان در سال 88-1387 به صورت تصادفي به دو گروه آزمون و شاهد تقسيم شدند. طراحي برنامه آموزشي مطابق با مراحل الگوي PRECEDE انجام شد. قبل از اجراي مداخله آموزشي، پرسش‌نامه‌هاي سنجش افسردگي، اضطراب و استرس (21DASS- یا Depression anxiety stress scale-21) و پرسش‌نامه‌هاي خود ساخته و استاندارد مطابق با مراحل الگوي PRECEDE براي دو گروه تكميل شد. گروه آزمون، مداخله مورد نظر را دريافت كردند (8 جلسه آموزشي، يك بار در هفته). سالمندان دو گروه در 2 ماه پس از مداخله آموزشي پيگيري شدند و دوباره پرسش‌نامه‌هاي مربوطه براي آن‌ها تكميل شد. داده‌هاي جمع‌آوري شده با استفاده از نرم‌افزار 15SPSS و آزمون‌هاي آماري t Independent، Paired t، 2χ، Wilcoxon و Mann-Whitney در سطح معنی‌داري 05/0 ≤ P مورد تجزيه و تحليل آماري قرار گرفت. یافته‌ها: تفاوت معنی‌داری در گروه آزمون نسبت به گروه شاهد در ميانگين نمرات عوامل مستعد كننده آگاهي (001/0 ≤ P) و نگرش (001/0 ≤ P)، عوامل قادر كننده (001/0 ≤ P)، عوامل مستعد كننده (001/0 ≤ P) و عملكرد به خصوص انجام مهارت‌هاي تنفس عميق و آرام‌سازي (001/0 ≤ P) وجود داشت. ميانگين امتيازات و شدت استرس (001/0 ≤ P) سالمندان نیز پس از اجراي مداخله معنی‌دار بود. نتیجه‌گیری: يافته‌ها كارايي، اثربخشي و تأثيرگذاري برنامه آموزشي بر اساس الگوي PRECEDE را جهت پيشگيري و كاهش ميزان استرس سالمندان تأييد کرد.

Title: بررسی ارتباط شاخص توانایی انجام کار (Work ability index) و ظرفیت فیزیکی انجام کار (Physical work capacity) بر اساس معادله فاکس (VO2 max) در کادر پرستاری مرد شاغل در بیمارستان‌های شهر اصفهان

http://hsr.mui.ac.ir/index.php/jhsr/article/view/465

مقدمه: ارزیابی صحیح توانایی کار شاغلین از نقطه نظر اقتصادی برای کارفرمایان حایز اهمیت است و تقویت آن یکی از راه‌های افزایش بهره‌وری منابع انسانی در صنایع و سازمان‌ها می‌باشد. هدف این مطالعه تعیین توانایی شاغلین با استفاده از دو شاخص ظرفیت فیزیکی انجام کار (PWC)، شاخص توانایی انجام کار (WAI) و تعیین همبستگی بین آن‌ها بود. تعیین شاخص PWC با توجه به مقادیر WAI جهت صرفه‌جویی در هزینه و زمان اندازه‌گیری دستگاهی PWC از اهداف دیگر طرح است. روش‌ها: پژوهش حاضر از نوع تحلیلی- مقطعی و بدون جهت است. افراد مورد مطالعه 228 نفر از کادر پرستاری بیمارستان‌های شهر اصفهان بودند که به صورت تصادفی ساده انتخاب شده‌اند. شاخص توانایی انجام کار با استفاده از پرسش‌نامه WAI و شاخص PWC بر اساس معادله فاکس و با استفاده از دوچرخه ارگومتر تعیین شد. سپس داده‌های مطالعه با استفاده از نرم‌افزار 16SPSS مورد تجزیه و تحلیل قرار گرفت.‌ یافته‌ها: میانگین شاخص WAI در افراد مورد مطالعه (4/4 ± 25/38) و میانگین ظرفیت فیزیکی کار PWC (7/0 ± 45/4) به دست آمد. نتایج آزمون Pearson نشان داد که بین ظرفیت فیزیکی انجام کار و WAI در گروه‌های سنی مختلف همبستگی معنی‌دار وجود ندارد (05/0 < P، 3/0 = r). تحلیل رگرسیون خطی چندگانه نشان داد که متغیرهای سن و بیماری‌های تشخیص داده شده توسط پزشک، تأثیرگذارترین عامل بر WAI می‌باشد (05/0 > ‌P‌، 18/0 = β). آزمون Pearson نشان داد که بین تعداد بیماری‌های تشخیص داده شده توسط پزشک و شاخص PWC در گروه‌های سنی 49-40 سال همبستگی معنی‌دار وجود دارد. نتیجه‌گیری: میانگین شاخص WAI این مطالعه مانند سایر مطالعات در مشاغل مشابه در سطح قابل قبول قرار داشت (36 < ‌‌WAI). شاخص WAI و PWC در گروه‌های سنی مختلف ارتباط همبستگی معنی‌دار را نشان نداد (05/0 < P، 3/0 = r)، این حاکی از آن است که تفاوت‌های اساسی در ارزیابی توانایی کار به وسیله دو شاخص وجود دارد و نمی‌توان با استفاده از مقادیر WAI شاخص PWC را پیش‌بینی کرد. با توجه به نتایج PWC و سطح فعالیت کادر پرستاری (سبک- متوسط) شاخص WAI ابزار مناسبی جهت تعیین توانایی شاغلین بود. بر اساس این مطالعه 6/27 درصد از افراد در معرض ریسک توانایی کار متوسط- ضعیف قرار داشتند (37 > ‌WAI)، که 8/1 برابر مقادیر مرجع انستیتوی بهداشت شغلی فنلاند است. این نسبت می‌تواند ناشی نوبت کاری و ساعات کار زیاد پرستاران باشد.

Title: جداسازی شوینده‌های آنیونی با فرآیند شناورسازی با هوای محلول از آب‌های سطحی

http://hsr.mui.ac.ir/index.php/jhsr/article/view/486

زمینه و هدف: توسعه صنایع و رشد جمعیت همراه با ارتقای سطح بهداشت، موجب به کارگیری وسیع مواد شوینده شده است. این مواد از طریق فاضلاب‌ها به آب‌های سطحی تخلیه شده، موجب مخاطرات زیست محیطی می‌شوند. هدف از این تحقیق، جداسازی شوینده‌های آنیونی از آب‌های سطحی توسط سیستم شناورسازی با هوای محلول می‌باشد. روش کار: یک واحد پایلوت شناورسازی با هوای محلول با جریان پیوسته شامل حوضچه انعقاد، لخته‌سازی و شناورسازی ساخته شد. منحنی كالیبراسیون با غلظت‌های استاندارد شوینده آنیونی تهیه شد. با مخلوط كردن نمونه‌های آب خام با سورفاكتانت آنیونی، آزمایشات در فشارهای اشباع‌سازی 3، 4 و 5 اتمسفر، غلظت‌های مختلف منعقد کننده از  mg/l5 تا 15 و در زمان‌های شناورسازی 3/19 و 26 دقیقه، انجام گرفت. نتایج: در کلیه نمونه‌های آزمایش شده pH آب خام بین 8 تا 2/8 و کدورت اولیه بین NTU 4/7 تا 144 بود. دوز اپتیمم ماده منعقد کنندهmg/l  15 به دست آمد. در دوزهای مختلفی از منعقد كننده بینmg/l  5 تا mg/l 15 و در زمان شناورسازی 26 دقیقه و 3/19 دقیقه، حداكثر راندمان حذف LAS در فشارهای مختلف به ترتیب 91 درصد و 1/41 درصد به دست آمد. آنالیزهای آماری رابطه معنی‌داری بین حذف کدورت با حذف شوینده‌های آنیونی نشان داد. نتیجه‌گیری: در این فرایند فاکتورهایی مانند فشار اشباع‌سازی، زمان شناورسازی و كاربرد ماده منعقد کننده در کارایی سیستم تأثیرگذار بوده است و حذف شوینده‌ها به دو طریق كف كردن و جذب بر روی ذرات فلوک سر سوزنی صورت می‌گیرد.
